# Quantitative gene expression assessment identifies appropriate cell line models for individual cervical cancer pathways

**DOI:** 10.1186/1471-2164-8-117

**Published:** 2007-05-10

**Authors:** Mark W Carlson, Vishwanath R Iyer, Edward M Marcotte

**Affiliations:** 1 Department of Biomedical Engineering, The University of Texas at Austin, Austin, Texas 78712, USA; 2 Center for Systems and Synthetic Biology, Institute for Cellular and Molecular Biology, The University of Texas at Austin, Austin, Texas 78712, USA

## Abstract

**Background:**

Cell lines have been used to study cancer for decades, but truly quantitative assessment of their performance as models is often lacking. We used gene expression profiling to quantitatively assess the gene expression of nine cell line models of cervical cancer.

**Results:**

We find a wide variation in the extent to which different cell culture models mimic late-stage invasive cervical cancer biopsies. The lowest agreement was from monolayer HeLa cells, a common cervical cancer model; the highest agreement was from primary epithelial cells, C4-I, and C4-II cell lines. In addition, HeLa and SiHa cell lines cultured in an organotypic environment increased their correlation to cervical cancer significantly. We also find wide variation in agreement when we considered how well individual biological pathways model cervical cancer. Cell lines with an anti-correlation to cervical cancer were also identified and should be avoided.

**Conclusion:**

Using gene expression profiling and quantitative analysis, we have characterized nine cell lines with respect to how well they serve as models of cervical cancer. Applying this method to individual pathways, we identified the appropriateness of particular cell lines for studying specific pathways in cervical cancer. This study will allow researchers to choose a cell line with the highest correlation to cervical cancer at a pathway level. This method is applicable to other cancers and could be used to identify the appropriate cell line and growth condition to employ when studying other cancers.

## Background

Cancer cell lines have been widely used as models of human cancer to better understand the biology of tumor formation and progression, as well as to help develop new therapeutic agents to treat the disease [1,2]. However, cell lines do not duplicate the *in vivo *environment, are subject to genetic drift, and cell-cell interactions are lost [3,4]. Therefore, we sought to quantitatively assess which of the commonly used cell lines in cervical cancer research were better models of cervical cancer relative to the cell lines we tested.

Current *in vitro *research of cervical cancer involves the culture of immortal cervical cell lines in monolayer [1,5]. Cell lines typically used include HeLa, SiHa, and Caski, among others. An alternative method to culturing in monolayer is organotypic culture, an advanced cell culture technique that transforms the growth environment from two dimensions into three dimensions. Organotypic culture imitates the *in vivo *phenotypic structure of epithelial tissue and has been used in different types of research, including breast [6,7], skin [8,9], cervical [10], and head and neck [11] cancer, as well as to study epithelial differentiation [12] and individual gene expression [10,13].

Few attempts have been made to quantitatively assess how closely cell lines actually model tissue. Most results from analyses such as clustering of expression profiles of cell lines and tissues simply conclude that cell lines resemble their tissue of origin [14]. For example, Sandberg *et al*. used the NCI 60 microarray data to compare cell lines in culture to their respective cancer microarray data sets. They performed a singular value decomposition (SVD) cluster analysis and generated a quantitative value termed the tissue similarity index (TSI), which denotes how well various cell lines still represent their tissue of origin [15].

With this in mind, we wanted to quantitatively assess how well cervical cancer cell lines commonly used to study cervical cancer actually model the disease, focusing on late-stage invasive cervical cancer. Therefore, the gene expression profiles of nine different cervical cell lines were correlated separately to the expression profiles of nine cervical cancer and three normal cervical biopsies. In addition to their American Type Culture Collection (ATCC) recommended culture media, HeLa and SiHa cell lines were additionally cultured in different media as well as in an organotypic environment to assess how their correlation to tissue changes in different culture environments. Conditions with higher correlations indicate better models of cervical tissue. In order to move beyond a simple global comparison, the correlations of each condition were also compared to cervical tissue at individual pathway-specific levels. This information provides a more detailed view of our ability to model cancer using cell lines.

## Results and discussion

### Differential gene expression between cervical cancer and normal cervix

Before comparing cervical cancer transcriptional profiles with cell line profiles, we first determined that the biopsies used in the analysis accurately represented cervical tissue and invasive cervical cancer. We tested the differentially expressed genes between normal and cancerous biopsies by first examining transcriptional changes in light of presumed biological mechanisms of cancer biology; we then compared the differentially expressed genes with regard to previous cervical cancer research.

We first used presumed biological mechanisms of cancer biology to assess our differentially expressed genes. First, groups of genes that represented common cancer pathways were checked for correct expression changes [16,17]. A t-test using the *Benjamini-Hochberg *multiple hypothesis correction (p < 0.01) and a 2-fold minimum expression change was used to identify 140 genes that were differentially expressed between cervical cancer and normal cervix. The 140 genes [see Additional file 1] were involved in many expected pathways involved in tumorigenesis; the top 16 up- and down-regulated genes can be found in Table 1. Individual genes, as well as sets of pathways including cell proliferation, cell-cell adhesion, and cell differentiation, were identified as correctly changing expression according to the general biology of cancer.

**Figure 1:**
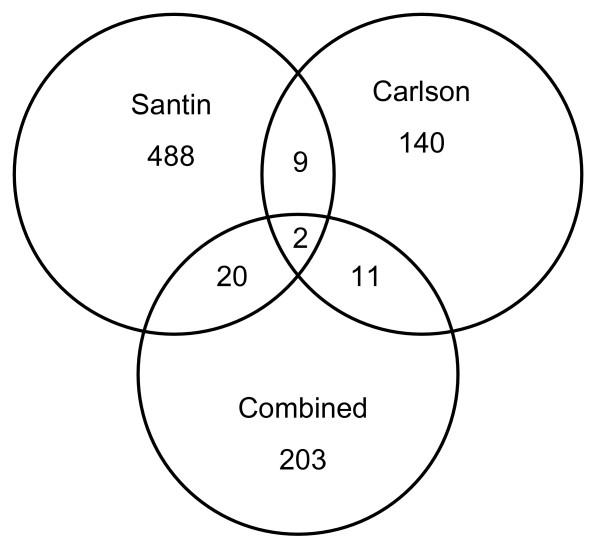
**Venn diagram of the overlap of differentially expressed cervical cancer genes in the literature**. Eight reports of differentially expressed cervical cancer genes from the literature were compared against each other and to our results. Seven of the studies were combined into one group (203 genes, no overlap between studies). Our results (Carlson, 140 genes) overlapped with Santin (488 genes) by 9 genes and with the rest by 11 genes. Santin had 20 genes in common with the 203 gene combined group. Only 2 genes were found in all three data sets, *SLC2A1 *and a serine protease inhibitor (clone IDs 25389 and 2562939). Our results show comparable overlap with the literature, and provide additional evidence that the tissue samples analyzed are representative of previous reports on cervical cancer.

A second analysis provided further evidence that the expression profiles in these cervical cancer biopsies were consistent with previous observations in the literature and therefore suitable for further detailed analysis. Approximately 650 differentially expressed genes identified in the literature, derived from small scale microarray studies [18-22], differential RNA display [23-27], or single gene studies [28,29], were compared to the 140 genes identified in our study (Figure 1). Nineteen genes were observed in both data sets (*) [see Additional file 1]; 30 genes in the same sequence family (homologs) were also identified (**) [see Additional file 1]. Despite the apparent small overlap in large scale datasets, the overlap is significant (p < 0.001, hypergeometric distribution) and indicates that our tissue biopsies are representative of the literature and can be used for further analysis.

**Figure 2:**
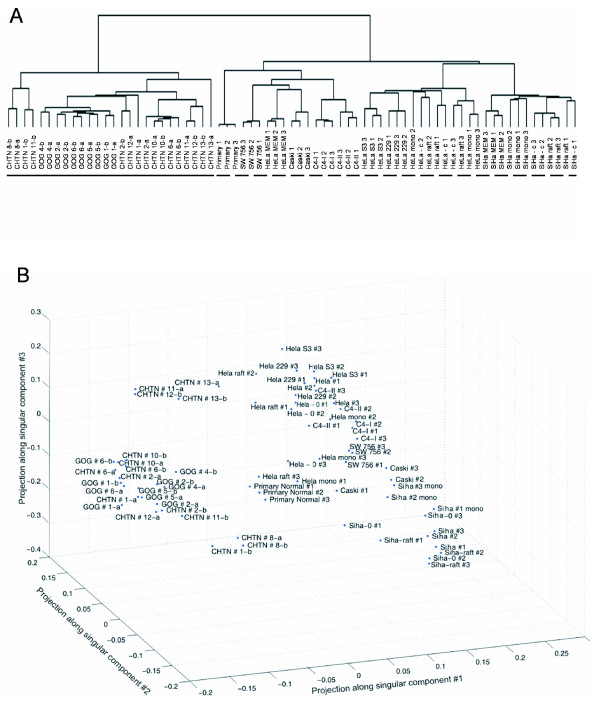
**Global transcriptional relationships among tissue samples and cell lines indicate high reproducibility of replicates**. A: Hierarchical clustering of samples by their expression profiles. Labels with "a" and "b" represent technical replicates, whereas numbered labels represent biological replicates. The primary separation occurs between cell lines (dashed bar) and cervical tissue (solid bar). All GOG samples and CHTN samples #1, 2, 8, 12 and 13 were invasive cervical cancer biopsies. CHTN samples #6, 10, and 11 were normal cervix. Most replicates clustered together, indicating the data was of high quality. Spots present on the microarray that had a median intensity over background of at least 150 and were present in 80% of the arrays were included in the analysis, resulting in 8,338 genes. B: Singular value decomposition of transcriptional profiles reveals general relationships among the samples, positioned here as the projections among the first 3 singular components (accounting for 40% of the variance, [see Additional file 6]. Again, cell lines were separated from cervical tissue.

A transcriptional comparison of cervical tissue to cell lines was performed using hierarchical clustering (Figure 2A) and SVD (Figure 2B) [30]. With few exceptions, all replicates were seen to cluster together, indicating high data quality. The few exceptions were likely due to small changes in gene expression, for example the organotypic and organotypic control (without fibroblasts) samples only exhibit small numbers of gene changes that are overshadowed when subjected to large scale clustering. Hierarchical clustering separated cell lines from tissue but did not provide information on how well cell lines model cervical cancer. A more quantitative assessment was used next to determine which cell line and growth condition most resembled cervical cancer at a pathway level.

We recognize that the particular cervical cancer biopsy samples that we used in this study do not represent the diversity of all possible cervical cancer samples. However, the comparisons described above provide strong evidence that the expression profiles collected accurately represent both cervical cancer and normal cervix. Once the reliability of the expression profiles was established, they could be used to compare the cell line expression profiles to identify which of the tested cell lines is a better model of cervical tissue.

### Global correlations of cell line models to cervical cancer

**Figure 3:**
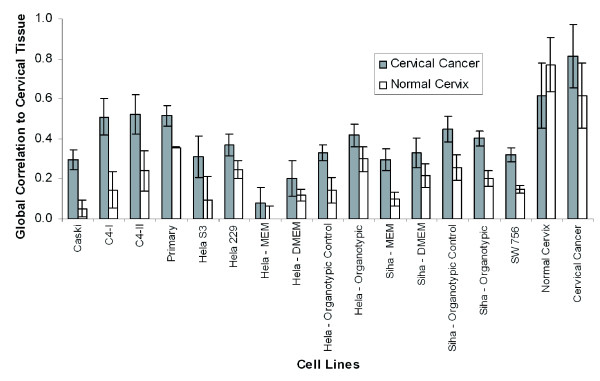
**Gene expression correlations quantitatively identify better cell line models of cervical tissue**. Gene expression correlations were calculated for all cell lines and growth conditions against both normal cervix and cervical cancer. Cell lines were cultured in ATCC recommended media as monolayers. SiHa and HeLa cell lines were also cultured in different media as well as in an organotypic environment. In addition to the organotypic culture, a control was used that left out the fibroblasts, which prevented the epithelial cell line to stack in 3-dimensions. The primary, C4-I, and C4-II cell lines had the highest correlation to cervical cancer and therefore were the better general models of cervical cancer out of the cell lines we tested. Changing the media from MEM to DMEM increased the correlation to cervical cancer for the HeLa and SiHa cell lines, as well as culturing them in an organotypic environment. Error bars were derived from the standard deviation of the correlation of a cell line against each individual patient biopsy.

The correlation of cell lines to both cervical cancer and normal cervix were calculated to evaluate how well different cell lines and culture conditions modeled the gene expression programs in cervical cancer. The global correlation of transcriptional profiles provided a quantitative assessment of how well cell lines model tissue; a higher Pearson correlation coefficient denoted a better model of cervical tissue. The correlations for each cell line and growth condition were summarized in Figure 3. All immortal cell lines were cultured in their ATCC recommended media [see Additional file 2]. To assess how changes to the environment affected the correlation between cell lines and cervical tissue, HeLa and SiHa cell lines were also cultured in a different type of media. These cell lines were individually cultured first in Eagle's Minimum Essential Medium (MEM) and later in Dulbecco's Modified Eagle's Medium (DMEM). In addition to the media change, HeLa and SiHa cell lines were cultured in an organotypic environment as well as an organotypic, fibroblast free control. The organotypic culture constructs a 3-dimensional growth environment, whereas the organotypic control simply results in a monolayer culture on a bed of collagen.

The correlation of normal to normal tissue or cancer to cancer tissue samples was calculated to provide a best-case scenario for the cell line correlations and to provide an estimate of patient variation. These results also provided a measure of how well cell lines can be expected to model tissue in general. The Pearson correlation coefficient among cervical cancer specimens was 0.81 while the correlation among normal cervix specimens was 0.83, setting the upper expected limit of this measure. The correlation of cervical cancer to normal cervix was higher than any cell line at 0.62. Of the cell lines we tested, the primary cell line was found to be a better model than others, of both cervical cancer and normal cervix in terms of overall mRNA expression correlation. The primary cell line was expected to have the highest correlation to cervical cancer over the other cell lines because it was more recently out of its *in vivo *environment. HeLa cultured as a monolayer had a surprisingly poor correlation to cervical cancer, given that it has been extensively used in cancer research [31-34]. HeLa was the poorest model of cervical cancer with a correlation of 0.08, consistent with HeLa cells' separation from other cell lines in the SVD analysis. However, HeLa cells increased their correlation to cervical cancer (0.42) when cultured in an organotypic environment, which was not evident in the SVD analysis. This analysis determined that relatively simple changes to a cell culture, such as different types of media, can affect how well a cell line can model tissue. The structural environment had a large impact on how well a cell line models the *in vivo *environment. It is possible that culturing the primary, C4-I, and C4-II cell lines in an organotypic environment would further increase their correlation to both cervical cancer and normal cervix.

Once we had identified a cell line with the highest correlation to cervical cancer (C4-I), we generated a list of 196 differentially expressed genes between the C4-I and primary normal cell lines. This list was used to hierarchically cluster the normal cervix and cervical cancer biopsies. The resulting dendrogram [see Additional file 3] demonstrates that potential biomarkers can be derived from cell lines that can separate the normal and cancer biopsies as well as their own complete expression signatures (Figure 2A). Cell lines can therefore be used to find potential biomarkers if it is known that they actually model the tissue reasonably well.

This quantitative analysis provided information on how changes to the culture environment can change a cell line's performance as a model to cervical cancer. To provide a deeper analysis of how well cell lines model tissue, the correlation of each Gene Ontology (GO) pathway was calculated to both cervical cancer and normal cervix for each cell line and growth condition.

### Pathway correlations of cell line models to cervical cancer

**Figure 4:**
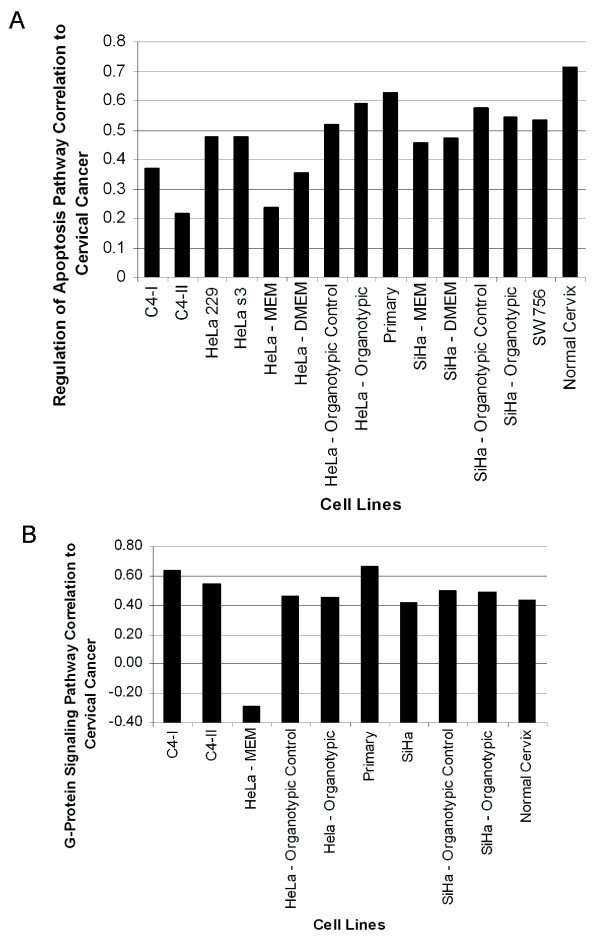
**Variation in the modeling performance of cell lines to cervical cancer at a pathway-specific level**. A: The "Regulation of Apoptosis" pathway (GO:42981) revealed that better global model to cervical cancer (primary, C4-I, and C4-II, discussed above) were not necessarily better models of specific pathways. In this pathway, the best models among those we tested were the primary cell line and HeLa cultured in an organotypic environment. Eight genes from the sub-pathway "Anti-Apoptosis" (GO:6916) were primarily responsible for lowering the correlation of the C4-II cell line. An average of 71 ± 5 genes were used to calculate the correlation for each cell line. B: In the example of the "G-protein Signaling" pathway (GO:7186), HeLa cultured as a monolayer in ATCC recommended media had an anti-correlation (-0.3) to cervical cancer. Fourteen out of 71 genes were primarily responsible for the negative correlation to cervical cancer. An average of 72 ± 7 genes were used to calculate the correlation for each cell line.

The global correlation of cell line models to tissue provided an analytical way to choose better overall models of cervical tissue; however, choosing an appropriate model for a specific pathway may be of more utility. Using GO annotations, the correlation for each pathway and for all cell lines and growth conditions was calculated against both cervical cancer and normal cervix. As shown in Figure 4, cell lines varied dramatically in the extent to which they modeled cervical cancer for a particular pathway. As discussed above, the better overall models of both cervical cancer and normal cervix that we tested were the primary, C4-I, and C4-II cell lines, with global correlations to cervical cancer of R = 0.52, 0.51, and 0.52, respectively. However, when studying the "Regulation of Apoptosis" pathway (GO:42981) for example, the primary cell line retained a high correlation (R = 0.62) but the C4-I and C4-II cell lines perform poorly with correlations of 0.37 and 0.22, respectively (Figure 4A). Further analysis of the genes that resulted in a lower correlation for the cell line C4-II resulted in 8 genes in the "Anti-Apoptosis" pathway (GO:6916), a sub-pathway of "Regulation of Apoptosis". These genes included *BAG1*, *BFAR*, *BIRC1*, *BIRC3*, *BIRC4*, *MALT1*, *PRKCZ*, and *TNFAIP3*, and had a 2–7 fold difference when compared to cervical cancer. This pathway failed to achieve significance when calculating individual pathway correlations, but was strong enough to lower the correlation for C4-II in the "Regulation of Apoptosis" pathway calculation. Interestingly, the HeLa cell line cultured in an organotypic environment had a correlation of 0.6 for the "Regulation of Apoptosis" pathway, whereas its global correlation to cervical cancer was 0.42. This pathway was not an exception, the organotypic HeLa cell line had a stronger correlation (R = 0.59) for six apoptotic pathways than even the primary cell line (R = 0.56).

Of even greater importance, some cell lines had negative pathway-specific correlations to cervical cancer. For example, HeLa cells cultured in monolayer had a negative (-0.3) correlation to cervical cancer in the pathway "G-Protein Signaling" (GO:7186) (Figure 4B). Fourteen out of 71 genes resulted in the negative correlation to cervical cancer, since they showed opposite expression to cervical cancer (>3-fold change). These genes included *GNG11*, *CXCL1*, *FZD2*, *GNA12*, *CALU*, *GPR19*, *AKAP12*, *GRINA*, 2 ESTs, *CALM2*, *GNAI2*, *DGKD*, and *EDNRB*, and had a 3–50 fold difference when compared to cervical cancer.

Researchers studying a particular gene or pathway may not be interested in the best global model if that cell line does not represent their pathway of interest. Only cell lines with the highest correlation should be used to study a specific pathway *in vitro*. The example where HeLa had a negative correlation to the "G-Protein Signaling" pathway is extremely important, indicating that this system is dysregulated in these cells relative to cervical cancer. Research on this pathway involving HeLa cells as a model may draw inconclusive results. Care must be taken to identify which cell line and growth condition would yield the most appropriate model. The results here can not be quantitatively represented by cluster analyses such as SVD. Therefore, the pathway analysis was of great use when determining which cell line should be used to model cervical cancer or normal cervix at a pathway-specific level.

### Highest and lowest pathway correlations to cervical tissue

**Figure 5:**
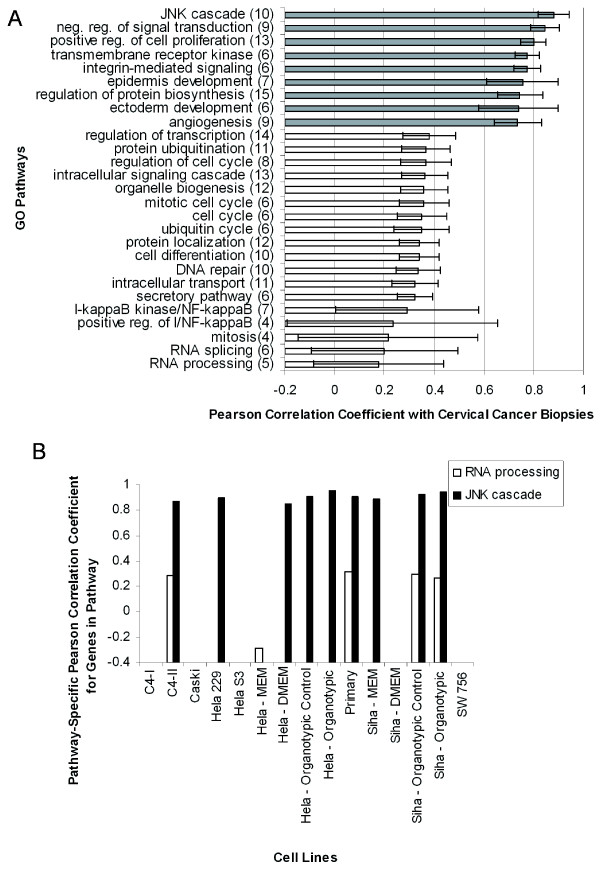
**Highest and lowest correlations of modeled GO pathways between cell lines and cancer, as well as normal cervix and cervical cancer**. A: The pathways where almost any cell line is an adequate model of either normal cervix or cervical cancer are shaded grey, while the pathways where only one or two cell lines are adequate models are white. The pathway example "RNA Processing" indicates some cell lines were anti-correlated and therefore a quantitative analysis was needed to identify better models that could be used to study this pathway. Error bars were generated from the correlation of a single cell line for each pathway and calculating the standard deviation. The pathways shown here represented a minimum of four cell lines or growth conditions. Numbers in parenthesis indicate how many cell lines were used to calculate the correlation. B: The highest and lowest pathway correlations between normal cervix and cervical cancer. The JNK cascade has a high correlation between normal and tumor, and is modeled well by most cell lines (Figure 5A). Mitosis and a number of other pathways involved in growth and regulation show poor correlation in their gene expression between normal and tumor, as expected. Numbers in parenthesis indicate how many genes were used to calculate the Pearson correlation coefficient.

In order to identify pathways that require more attention when selecting a cell line model, the correlations of all cell lines to cervical cancer for a specific pathway were averaged to generate a single correlation that represented all cell lines and growth conditions (Figure 5A). Biologically relevant pathways that had the highest and lowest correlation to tumor tissue were plotted. Examples include the JNK cascade, which was modeled well by all cell lines, whereas the "RNA Processing" pathway and several cell cycle pathways were the most poorly represented by most cell lines, compared against cervical cancer. These examples are plotted in detail in Additional file 4.

A pathway analysis of normal cervix versus cervical cancer is shown in Figure 5B. Pathways that share similar gene expression between normal and cancer include the regulation of cytokines, the JNK cascade, and a few metabolic pathways; the JNK cascade is modeled well in most cell lines studied as mentioned above (Figure 5A). Pathways with a low correlation between normal and tumor cervical tissue include mitosis, G-protein signaling, and regulation of development. The "mitosis" pathway is modeled poorly by most cell lines when compared to cervical cancer (Figure 5A), and has a low correlation between normal and tumor tissue (Figure 5B). Further, the correlation to normal tissue by most cell lines is -0.3, indicating that mitosis is poorly modeled by cell lines in general, but model tumor tissue much better than normal tissue, as expected.

Most of these cell lines have been outside the *in vivo *environment for decades and as a consequence have adapted to their new environment, resulting in changes of gene expression. We expected to see many important pathways with low correlations to tissue, and this was the case. We observed poor correlation to the cell cycle, RNA processing, and cell signaling pathways in cell lines compared to cervical cancer. This was due perhaps to both accumulated mutations and the fact that cultured cell lines may have different modes of cell-to-cell communication. These pathways, with low correlation to tissue (typically below 0.3), are extensively studied in cancer research [35-37]. There were pathways that retained a high correlation to tissue across many cell lines, including the JNK cascade, positive regulation of cell proliferation, and other transcriptional regulation pathways, which indicates they are still relevant to study by researchers using current cell lines.

### Media effect on correlation

**Figure 6:**
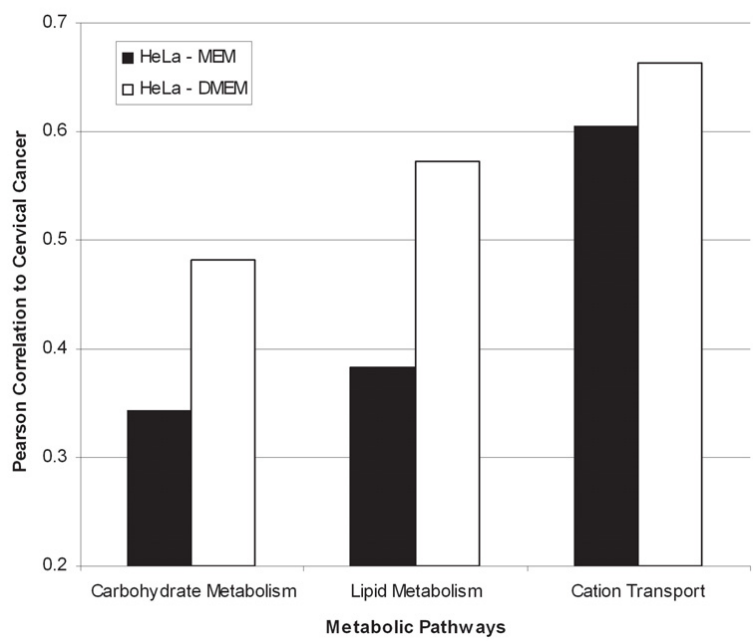
**Simple media changes to culture conditions increase the HeLa cell line's correlation to cervical cancer**. Three pathways provided examples of how the correlation to normal cervix increased when HeLa cells were cultured in rich DMEM media versus ATCC recommended MEM media as monolayers. DMEM media contained glucose, and the expression of *PDK4 *indicated HeLa cells cultured in DMEM experience a nutrient-rich environment, similar to *in vivo *conditions.

HeLa and SiHa cell lines were cultured in a different type of media to assess whether small changes to the environment could have a large impact on the correlation to tissue. The effect of different culture media on the correlation to cervical tissue was assessed at the pathway level using both SiHa and HeLa cell lines. An example of three biologically relevant pathways that changed in correlation between the two media is shown in Figure 6, though the changes were not restricted to metabolic pathways. The correlation to cervical cancer increased by changing the medium to DMEM; the increased correlation to cervical tissue was expected after the transition from a minimal medium (MEM) to a richer medium (DMEM). We hypothesized that the increase in correlation was due to the addition of glucose in DMEM, which is absent from MEM. After ranking the genes based on their impact on the correlation, the third highest gene that lowered the correlation of HeLa cultured in MEM was *PDK4*. Pyruvate dehydrogenase kinase has been found to increase its expression during starvation [38]. In our experiments, *PDK4 *was found to have a 4-fold decrease in expression in cervical cancer, a 2-fold increase in expression in HeLa cultured with DMEM, and a 20-fold increase in expression in HeLa cultured with MEM. As the function of *PDK4 *is the regulation of glucose metabolism, this indicates HeLa cultured in MEM experience starvation-like conditions, whereas HeLa cultured in DMEM have an environment similar to *in vivo *cancer conditions. This example of *PDK4*, along with the three metabolic pathways shown in Figure 6, provided examples of how an increase in the correlation to normal cervix was achieved by simply changing the medium. Simple changes to the culturing environment can therefore have a dramatic affect on the relevance of some cell lines as models of cervical cancer.

### Organotypic effect on correlation

Organotypic culture physically resembles the structure of cervical epithelium. HeLa and SiHa cell lines increased the correlation to tissue when cells were cultured in organotypic cultures versus monolayer. The organotypic control, which consists of the same culture environment minus the fibroblasts, also increased the correlation over the cell lines, indicating a simple collagen bed was sufficient to increase the global modeling of cell lines to cervical cancer. In the case of the organotypic and organotypic control, the two environments did not differ significantly in their correlations (Figure 3).

**Figure 7:**
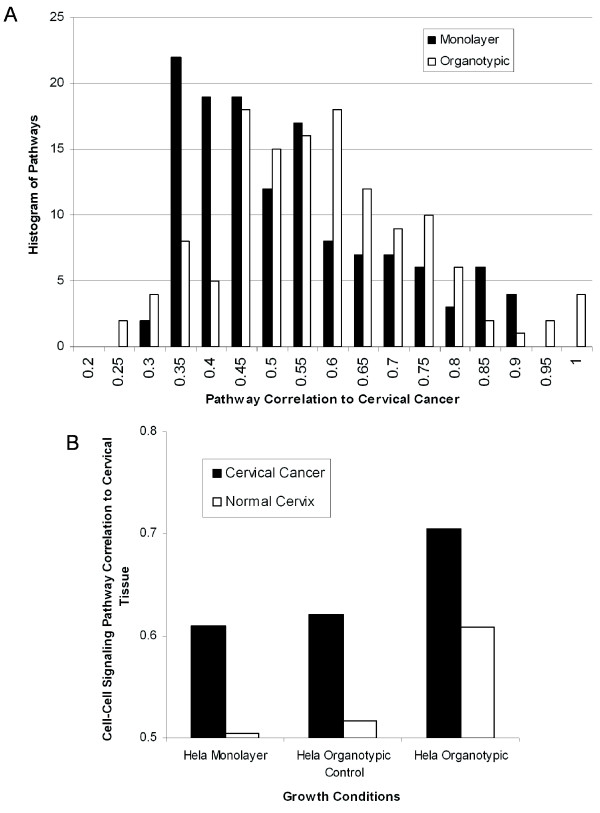
**HeLa organotypic cultures are better models of cervical cancer than HeLa monolayer cultures**. A: Histogram of the number of pathways at each specific correlation for HeLa cells cultured as monolayer (black) and in an organotypic environment (white). There were more pathways with a higher correlation to cervical cancer in the organotypic culture than in monolayer (p < 0.004, t-test). Therefore, organotypic cultures were better models of cervical cancer than simple monolayer cultures. B: In the ''Cell-Cell Signaling'' pathway (GO:7267), HeLa cells cultured in an organotypic culture had a higher correlation to both normal cervix and cervical cancer than either monolayer or organotypic control cultures.

Further evidence that organotypic cultures were better models over monolayer culture was provided by calculating the overall shift in pathway correlations for different environments, plotted as a histogram in Figure 7A. Since organotypic cultures had a high correlation to tissue, we expected to see an increase in the overall number of pathways that had a higher correlation to tissue by the organotypic culture. This was observed by a shift of the histogram to higher correlation and confirmed statistically (p < 0.004, t-test). Although significance was not reached when compared to normal cervical tissue, likely due to low sample numbers, an increase in the number of pathways with a higher correlation was still evident.

The individual gene expression changes between monolayer and organotypic environments was studied to shed light on how expression changes of a relatively few genes can affect their correlation to cervical cancer. For example, in the case of SiHa cells cultured as monolayer versus organotypic, we observed specific induction of membrane proteins. Cadherin, a cell adhesion gene, had a 3.6 fold increase in expression in the 3-dimensional culture over the monolayer, arguing for cadherin's role in stimulating cell stacking in the organotypic model. Interestingly, the cell adhesion gene *CYR61 *had a dramatic decrease (7 fold) in expression. There was an increase in expression in many genes whose function was integral to the plasma membrane, including *SLC7A11*, *SLC04A1*, *CLDND1*, *IER3*, *HOMER1*, and *AOC3*. Many of these genes play a role in metabolic signaling or plasma membrane transport and were possibly up-regulated due to the increased communication between cells in this 3-dimensional culture. Many of the gene changes that allowed a cell line to grow in 3-dimensions were involved in cell-cell signaling pathways (Figure 7B), further highlighting the importance of this pathway in tumorigenesis.

As cell-cell attachments and signaling play an important role in differentiation of the epithelium, the pathway "Cell-Cell Signaling" (GO:7267) was analyzed as to whether 3-dimensional culture conditions can improve the modeling performance of cell lines (Figure 7B). The "Cell-Cell Signaling" pathway contains 507 genes that transfer information from one cell to another. Some genes in this pathway include fibroblast growth factor, gap junctions, interleukins, and leptin precursors. The HeLa gene expression in the "Cell-Cell Signaling" pathway was more similar to both normal cervix and cervical cancer when cultured in the organotypic environment versus monolayer as well as the organotypic control. Therefore, HeLa organotypic experiments appear better models of cervical cancer than HeLa cultured in monolayer. The addition of fibroblasts allowed the formation of cell layers, which increased the cell-cell contact. This increase in contact apparently changes the communication between cells and thus increases the consistency in expression patterns of genes involved in cell-cell communication.

## Conclusion

Expression profiles of cervical cancer biopsies were compared to previous cervical cancer research to provide evidence that the expression profiles accurately represent both cervical cancer and normal cervix. Primary normal cells and the C4-I and C4-II cells lines were found to be better models than the other cell lines we tested, even the more commonly used HeLa cell line. We found that simple changes to the environment, such as media, increased the correlation of HeLa and SiHa cells to cervical cancer. In addition, culturing HeLa and SiHa cell lines in an organotypic environment rather than in monolayer significantly increased their correlation to cervical cancer. The correlation of each cell line and growth condition was also analyzed at the pathway level. Despite the fact that many cell lines still retain a high expression correlation to cervical cancer, our pathway level analysis also revealed cell lines that had an anti-correlation to cervical cancer. Cell lines with low correlations to cervical cancer should be avoided in future studies as models of this disease.

## Methods

### Monolayer cell culture

Nine cell lines were cultured in monolayer as well as under various perturbations, such as different media and structural environments. All cell lines except the primary normal epithelial line were obtained from the ATCC and cultured in ATCC recommended media. In addition to the recommended media, HeLa and SiHa cells were also grown in DMEM to assess how media changes their correlation to cervical cancer. Cell lines were cultured in 10% fetal bovine serum (FBS) (ATCC) and 1:100 PenStrep (Invitrogen). The primary cell line was a gift from Dr. Rebecca Richards-Kortum's lab at Rice University, and was grown in basal media and growth factors (cc-3118, Clonetics). All cell lines were cultured three times independently across three separate passages except the primary line, which was cultured at the same time in three separate plates due to the cells' short life spans. The cell line media conditions are summarized in Additional file 2. The replicates were individually hybridized to microarrays.

### Organotypic cell culture

Organotypic cultures consisted of NIH 3T3 fibroblasts, collagen, and an epithelial cell line. Collagen was prepared by adding 2.2 ml of type I rat tail collagen (3 mg/ml) (Roche), 220 μl of 10 × DMEM, 220 μl of FBS, and Hepes-NaOH for a final pH of 7.2. Fibroblasts were resuspended in prepared collagen at a cell density of 3 × 10^5^/ml in 3 ml of collagen. 120 μl of the fibroblast/collagen suspension was added to each transwell plate (3 μm pore size and 6.5 mm diameter) (Corning) and incubated for 30 minutes at 37°C to solidify the collagen. SiHa and HeLa cells were resuspended at a concentration of 1 × 10^6^/ml in DMEM media and 80 μl were added on top of the solid fibroblast/collagen suspension. 600 μl of DMEM media was added to the outside of the transwell insert and was replaced every other day. The organotypic control cultures were treated identically to the organotypic cultures, but without adding fibroblasts to the collagen; the absence of fibroblasts prevented 3-dimensional growth of the epithelial cell line.

### Cervical tissue

Three normal and nine moderately to poorly differentiated, invasive cervical cancer biopsies were obtained from the Cooperative Human Tissue Network (CHTN) and the Gynecologic Oncology Group (GOG) with the approval of the IRB at The University of Texas at Austin. Two technical replicates from each patient biopsy were hybridized separately to microarrays.

### mRNA isolation and amplification

Total RNA was isolated using Trizol (Invitrogen). RNA quality and quantity was assessed by gel electrophoresis and UV spectroscopy. DNA contamination was removed by RNeasy MinElute Cleanup (Qiagen). Universal Human Reference (UHR) RNA (Stratagene) was used as the reference channel in all hybridizations. RNA was amplified using T7 Message Amp (Ambion).

### cDNA microarrays

Microarrays were printed on poly-L lysine coated slides with 47,000 previously sequence-verified IMAGE clones (Research Genetics/Invitrogen) on each slide, as described by Gu and Iyer [39]. The reference channel consisted of 4 μg of amplified UHR. The experimental samples consisted of 4 μg of amplified RNA from cell lines or biopsies. Amino-allyl labeling and hybridization protocols were performed as previously described [39]. Samples were hybridized for 16 hours in the dark in humidity chambers (Corning). Slides were then washed, dried, coated in DyeSaver (Genisphere) and scanned with Axon 4000B GenePix scanners (Axon) at wavelengths of 532 nm for Cy3 and 635 nm for Cy5. Additional file 5 provides the day of hybridization, print set, sample number, and cluster dendrogram for all microarray experiments.

### cDNA microarray data analysis

Microarray images were processed using GenePix 4.0 software (Axon). After aligning the settings file and collecting the pixel intensity, the data were uploaded to the Longhorn Array Database (LAD) [40] for spot filtering and normalization. Significance testing was performed with Acuity 4.0 (Axon). In LAD, spots that were flagged during manual gridding or spots that had less than a median intensity of 150 were excluded from further analysis. After log_2_-transformation and background subtraction, data were normalized to a median intensity ratio of one. Only 8,338 genes with expression measurements on at least 80% of arrays were analyzed further. Averaged linked clustering and data centering was performed with LAD.

Calculation of the Pearson correlation between cell lines and tissue included the same data filtering described above; in addition, genes were also excluded if they were not present in at least 2 out of 3 cell line replicates. Replicates were averaged before calculating the correlation.

Two separate SVD analyses were performed, first on all samples and second solely on the cervical tissue to identify genes differentially expressed between normal cervix and cervical cancer. In both cases, SVD was performed on the 8,338 genes used for hierarchical clustering where the non-missing row average for a gene replaced any missing data for that gene. The columns (cell lines or tissue biopsies) were normalized. 499 genes were identified as differentially expressed between cervical cancer and normal cervix by rank ordering the genes according to fold change, and selecting genes with a greater than 2-fold change.

A Student's t-test was performed on the 8,338 genes described above to identify differentially expressed genes between cervical cancer and normal cervix. 434 genes were identified using a *Benjamini-Hochberg *multiple hypothesis correction (p < 0.01) with a minimum 2-fold expression change. The overlap of the SVD and t-test data produced a highly confident gene list of 140 genes. Additionally, a t-test was also used to identify 77 differentially expressed genes between SiHa cells grown as a monolayer versus an organotypic environment (p < 0.001).

The Pearson correlation coefficient was calculated between each cell line and, individually, against cervical cancer and normal cervix. Genes were subjected to the same filters used in the SVD analysis. Tissue to tissue comparisons were calculated by averaging each replicate and then splitting the patient samples into two groups.

The same data used to calculate the global correlation were used to calculate the pathway specific correlation. Replicates were averaged and the clone identifiers were mapped to LocusLink identifiers using SOURCE [41]. Clone IDs with more than 4 LocusLink identifiers were removed; the rest were annotated to Gene Ontology, Biological Process (levels 5–11) using LocusLink. A strict *Bonferroni *multiple hypothesis correction was used (p < 0.0005, t-test) based on the database size of GO.

## Authors' contributions

MWC carried out the cell culture, tissue procurement, mRNA isolation/amplification, and microarray hybridizations. VRI provided the construction of the microarrays and tools for the statistical analysis. MWC and EMM conceived the study and participated in its design and helped draft the manuscript. All authors read and approved the final manuscript.

## Supplementary Material

Additional file 1 The list of 140 genes differentially regulated between normal cervix and invasive cervical cancer. The intersection of a t-test using the *Benjamini-Hochberg *multiple hypothesis correction (p < 0.01) and the SVD results, along with a 2-fold minimum expression change, was used to identify 140 differentially expressed genes. 19 genes marked with an (*)were also reported in the cervical cancer literature. 30 genes marked with (**)were homologs that were reported in the previous cervical cancer literature. Click here for file

Additional file 2 Cell line culture conditions. Cell lines were cultured as monolayers in ATCC recommended media. Also, SiHa and HeLa cells were cultured in DMEM as well as in an organotypic environment. Click here for file

Additional file 3 Potential biomarkers derived from cell lines cluster normal cervix from cervical cancer in a manner comparable to the tissue-derived expression profiles. We identified a cell line with the highest correlation to cervical cancer (C4-I) and generated a list of 196 differentially expressed genes between the C4-I and primary normal cell lines. This list was used to hierarchical cluster the normal cervix and cervical cancer biopsies. The resulting dendrogram was similar to the dendrogram in Figure 2A. Click here for file

Additional file 4 JNK cascade is a well modeled pathway of cervical cancer by most cell lines whereas the RNA processing pathway is poorly modeled by most cell lines. A detailed view of two specific pathways indicate some pathways are well represented by many cell lines ("JNK Cascade"; GO:7254), whereas other pathways ("RNA Processing"; GO:6396) are poorly modeled by many cell lines. Missing data indicates significance was not reached for a particular cell line and not a correlation of -0.4. Click here for file

Additional file 5 Presentation of the day of hybridization, microarray print batch (written "HS-print batch-slide number"), and dendrogram. In order to reduce the chance of non-biological artifacts that may result in high correlations between samples, replicates for the most part were hybridized on different days. In addition, many samples, including tissue biopsies, were run on different print sets and different days. Additional file 3 presents the day of hybridization, print set, and dendrogram. Clusters on the dendrogram span print sets and days of hybridization, indicating high correlations observed in the analysis result from biological similarity and not technical artifacts. Click here for file

Additional file 6 Percent of SVD component variance. The first 3 components plotted in Figure 2B account for approximately 40% of the variance. SVD was calculated using Matlab. Click here for file

## Figures and Tables

**Table 1:** The top 16 up-and down-regulated genes between normal cervix and cervical cancer.

**Clone ID**	**Unigene ID**	**Gene Name**	**Gene Description**	**Fold Change**
810131	Hs.2785	KRT19	keratin 19**	21.2
135975	Hs.642947	DSP	desmoplakin*	14.8
2497698	Hs.156346	TOP2A	topoisomerase (DNA) II alpha*	13
2541366	Hs.234642	AQP3	aquaporin 3**	11.8
177074	Hs.591205	ZIC2	Zic family member 2	11.8
2444518	Hs.465506	PPAP2C	phosphatidic acid phosphatase type 2C	11.1
511428	Hs.301350	FXYD3	FXYD domain containing ion transport regulator 3	10
951241	Hs.615092	NUSAP1	nucleolar and spindle associated protein 1	9.5
712505	Hs.334562	CDC2	cell division cycle 2, G1 to S and G2 to M**	9.4
781089	Hs.350966	PTTG1	pituitary tumor-transforming 1*	9.3
25389	Hs.473721	SLC2A1	solute carrier family 2*	8.9
296155	Hs.434886	CDCA5	cell division cycle associated 5**	7.1
253009	Hs.556259	PRO1073	PRO1073 protein	6.6
378365	Hs.520421	PIGPC1	p53-induced protein PIGPC1	6
769921	Hs.93002	UBE2C	ubiquitin-conjugating enzyme E2C*	5.9
685026	Hs.481208	AMMECR1	Alport syndrome, midface hypoplasia	5.5
344139	Hs.104672	DOC1	downregulated in ovarian cancer 1	-6.7
813823	Hs.406475	LUM	lumican	-6.8
1607286	Hs.8867	CYR61	cysteine-rich, angiogenic inducer	-6.9
32609	Hs.213861	LAMA4	laminin, alpha 4**	-7
855557	Hs.472831	PKIG	protein kinase (cAMP-dependent) inhibitor gamma	-7
366100	Hs.189445	MATN2	matrilin 2	-7.2
2495781	Hs.516578	TFPI	tissue factor pathway inhibitor**	-7.3
878182	Hs.212838	A2M	alpha-2-macroglobulin	-8
300015	Hs.597358	LEPR	leptin receptor	-8.7
809719	Hs.477128	URB	steroid sensitive gene 1	-9.2
186757	Hs.591469	DDR2	discoidin domain receptor family	-9.8
49354	Hs.620557	ANK2	ankyrin 2, neuronal	-10.4
1534435	Hs.104839	TIMP2	tissue inhibitor of metalloproteinase 2	-10.7
839623	Hs.69855	NRAS	neuroblastoma RAS viral (v-ras) oncogene	-12.6
1636447	Hs.83381	GNG11	guanine nucleotide binding protein**	-15.8
813266	Hs.598421	FHL1	four and a half LIM domains 1	-21.6

A subset of the 140 genes differentially regulated between normal cervix and invasive cervical cancer, as shown in Additional file 1. Genes marked with an (*) were also reported in the cervical cancer literature; genes marked with an (**) were homologs that were reported in the previous cervical cancer literature.
