# Supplementary figures and images for: Quantitative gene expression assessment identifies appropriate cell line models for individual cervical cancer pathways
Source: BMC Genomics. 2007 May 10;8:117. doi: 10.1186/1471-2164-8-117 (PMC1878486; doi:10.1186/1471-2164-8-117)

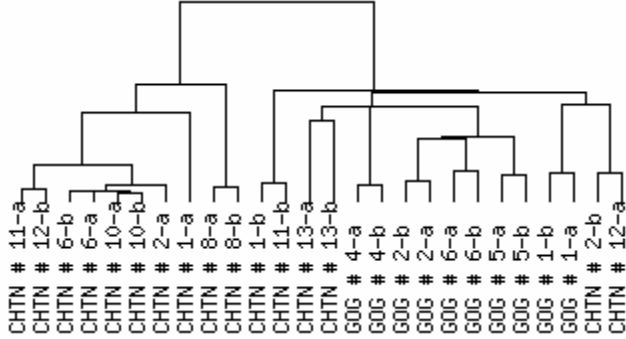

Supplement: Additional file 3 — Potential biomarkers derived from cell lines cluster normal cervix from cervical cancer in a manner comparable to the tissue-derived expression profiles. We identified a cell line with the highest correlation to cervical cancer (C4-I) and generated a list of 196 differentially expressed genes between the C4-I and primary normal cell lines. This list was used to hierarchical cluster the normal cervix and cervical cancer biopsies. The resulting dendrogram was similar to the dendrogram in Figure 2A. [file 1471-2164-8-117-S3.pdf]

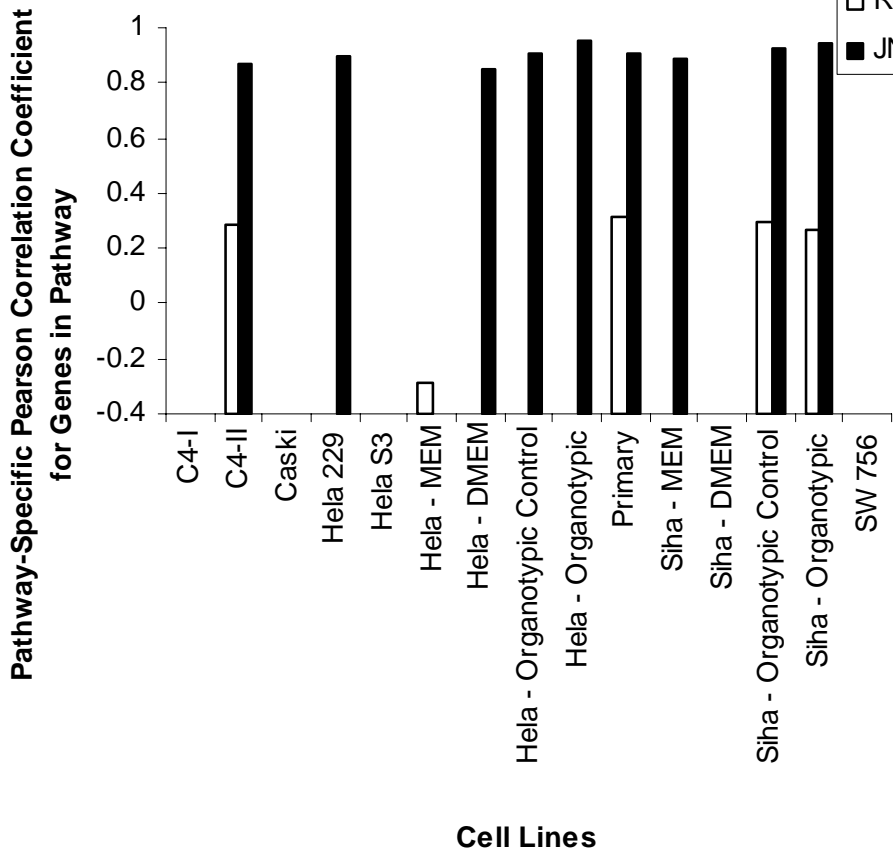

Supplement: Additional file 4 — JNK cascade is a well modeled pathway of cervical cancer by most cell lines whereas the RNA processing pathway is poorly modeled by most cell lines. A detailed view of two specific pathways indicate some pathways are well represented by many cell lines ("JNK Cascade"; GO:7254), whereas other pathways ("RNA Processing"; GO:6396) are poorly modeled by many cell lines. Missing data indicates significance was not reached for a particular cell line and not a correlation of -0.4. [file 1471-2164-8-117-S4.pdf]

Eigen Probabilities

Percent variance explained

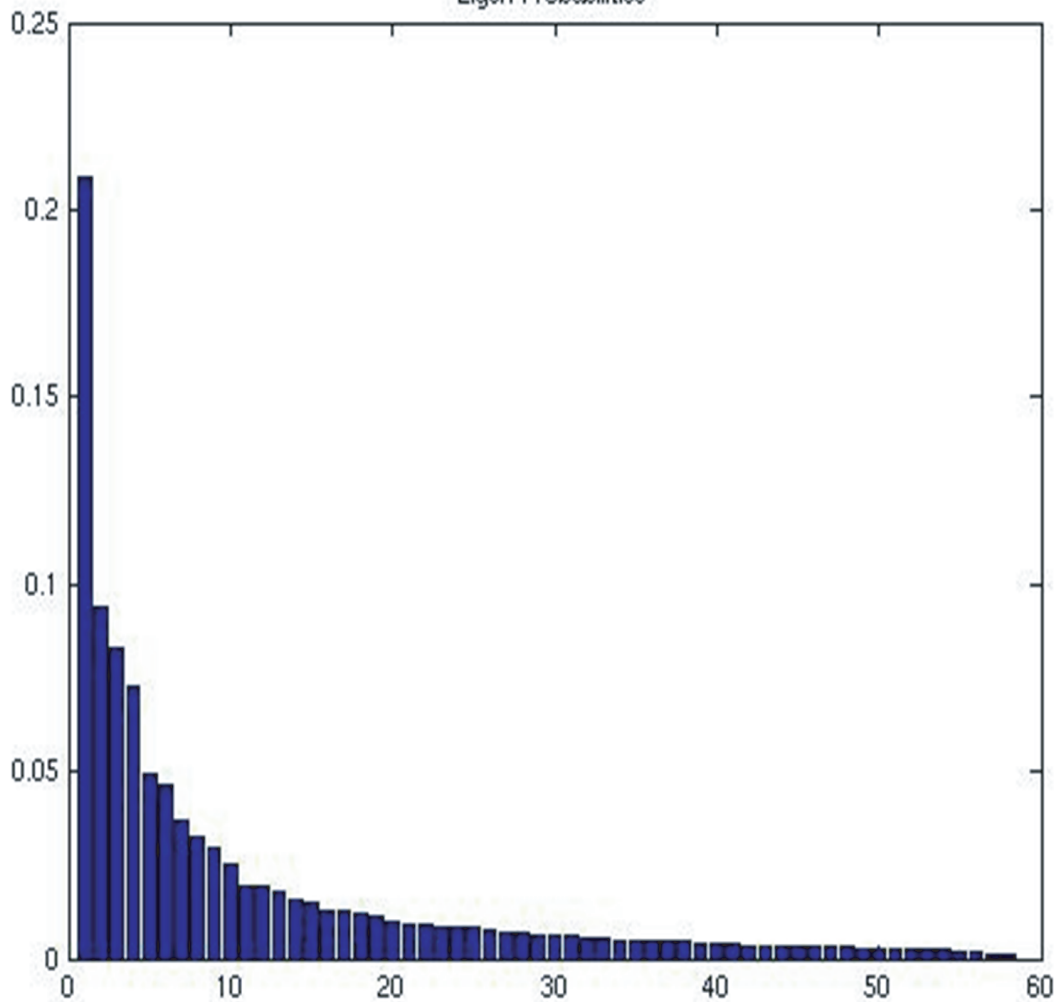

Singular component vectors

Supplement: Additional file 6 — Percent of SVD component variance. The first 3 components plotted in Figure 2B account for approximately 40% of the variance. SVD was calculated using Matlab. [file 1471-2164-8-117-S6.pdf]
